# Flammability and Thermal Properties of Rigid Polyurethane Foams Modified with Waste Biomass and Ash

**DOI:** 10.3390/ma18194570

**Published:** 2025-10-01

**Authors:** Anna Magiera, Monika Kuźnia, Rafał Stanik, Katarzyna Kaczorek-Chrobak, Maik Gude, Bartłomiej K. Papis

**Affiliations:** 1 AGH University of Krakow, Faculty of Metals Engineering and Industrial Computer Science, Department of Heat Engineering and Environment Protection, al. A. Mickiewicza 30, 30-059 Krakow, Poland; kuznia@agh.edu.pl; 2 TUD Dresden University of Technology, Institute of Lightweight Engineering and Polymer Technology, Holbeinstr. 3, 01307 Dresden, Germany; rafal.stanik@tu-dresden.de (R.S.); maik.gude@tu-dresden.de (M.G.); 3 Building Research Institute, Fire Research Department, ul. Filtrowa 1, 00-611 Warsaw, Poland; k.kaczorek-chrobak@itb.pl (K.K.-C.); b.papis@itb.pl (B.K.P.)

**Keywords:** rigid polyurethane foam, biomass, ash, biomass combustion, waste management, composite material, flammability, cone calorimetry, thermal properties, thermal analysis

## Abstract

The increasing demand for sustainable construction materials has driven interest in utilizing waste biomass within polymer composites. Rigid polyurethane foams, widely valued for thermal insulation, exhibit a significant flammability issue. This study investigates the impact of incorporating various waste biomass materials, including brewers’ spent grain, coffee grounds, and soybean husk and their combustion ashes on the selected properties of rigid polyurethane foams. The primary objective is to assess the potential of these eco-friendly additives as replacements for traditional raw materials, aiming to enhance fire resistance and thermal stability and thereby promoting circular economy principles in the construction sector. Composite foam samples were fabricated using a mixing and casting technique, incorporating 5% wt. of fillers into the polymer matrix. Thermal stability and flammability were evaluated using cone calorimetry and thermogravimetric analysis. The findings indicated that while biomass inclusion did not significantly improve char formation, the addition of ash substantially increased char yield, a critical factor in fire suppression. Although biomass and ash may influence flammability, they do not inherently bolster the intrinsic thermal stability of the polyurethane matrix itself.

## 1. Introduction

Insulating materials employed in the building industry exhibit a diverse range of thermal and flammability characteristics, impacting both energy efficiency and fire safety. Generally, organic insulation materials such as polystyrene and polyurethane foams offer excellent thermal performance, attributed to their low thermal conductivity [1]. For instance, expanded polystyrene (EPS) is primarily composed of air, contributing to its insulating properties, and modified polyurethane foams can achieve thermal conductivity values as low as 0.022 W m^−1^K^−1^ [2]. However, these materials are often highly combustible, exhibiting rapid ignition and flame spread, posing a significant fire hazard and releasing toxic gases upon combustion [1]. This necessitates the integration of flame retardants to enhance their fire-resistant properties [3].

In contrast, inorganic insulating materials like mineral fiber (including glass and basalt wool) are renowned for their superior fire resistance, often classified as non-combustible [4]. While they provide excellent fire protection, their thermal insulation performance can sometimes be lower, and their absorbability to moisture can further compromise their insulating capabilities, as water significantly increases thermal conductivity [1]. Phenolic foams, a type of organic foam, stand out for their exceptional fire properties, including high flame resistance and low smoke production [5]. Additionally, emerging bio-based materials, such as those derived from cork, straw bale, or hemp, are being explored as sustainable alternatives, offering promising thermal properties, though their fire behavior, including smoldering combustion, requires careful consideration and development for optimal performance in construction applications [6,7,8]. The overall objective in material selection involves balancing these thermal and flammability attributes to meet stringent building codes and enhance both energy efficiency and occupant safety.

The pursuit of sustainable and eco-friendly materials in construction and other industries has led to increased interest in utilizing waste biomass as modifiers in polymer composites [9]. Rigid polyurethane foams (RPUF) are widely employed as thermal insulating materials [10], but their inherent flammability poses a significant challenge [11]. To address this, researchers are exploring the incorporation of waste biomass, along with ash derived from their combustion, to enhance the fire resistance and thermal stability of these foams [9]. The rationale behind this approach is multifold: it diverts waste from landfills, reduces reliance on conventional flame retardants, and potentially improves the overall performance of the resulting polyurethane foam [9]. It is crucial to consider the fire behavior of crop-based insulation materials in both flaming and smoldering combustions, rather than solely focusing on their thermal performance [8]. Specifically, the introduction of lignocellulosic biomasses into the polyurethane (PU) matrix can potentially lead to a reduction in the heat release rate during combustion and an increase in char formation, effectively improving the fire resistance of the composite material [2,12,13].

The growing awareness of environmental issues and the need for sustainable practices has spurred research into bio-based and waste-derived materials as alternatives to conventional, petroleum-based components in PU formulations [14]. Incorporating waste biomass along with ash from their combustion, into RPUF represents a promising avenue for enhancing fire resistance and thermal stability while promoting circular economy principles [15,16]. This approach not only addresses waste management concerns but also has the potential to improve the overall performance and environmental footprint of PU foam products [9].

Brewers’ spent grain, coffee grounds, and soybean husks are byproducts of various industries [11]. These waste materials are generated in substantial quantities globally, and their disposal poses environmental and economic challenges [17]. Valorizing these waste streams by incorporating them into PU foams can provide a sustainable alternative to traditional fillers and additives. Ash, often a byproduct of combustion processes, is rich in minerals and can serve as a filler to improve the mechanical properties and thermal stability of polymer matrices [18]. The use of natural fillers in polymer material production has seen a sharp increase, driven by the low cost of biomass-derived raw materials and the desirable properties of the resulting polymer materials [19].

Brewers’ spent grain (BSG), a major byproduct of the brewing industry, presents several avenues for practical application. Given its high fiber and protein content [20,21], BSG can be repurposed as a valuable animal feed, offering a sustainable alternative to traditional feed sources. Additionally, BSG can be processed and incorporated into human food products, enhancing their nutritional profile [22]. Moreover, research explores its potential in biofuel production, harnessing its organic matter for renewable energy [23,24]. In the context of modifying polymeric materials, ground BSG can be integrated to potentially enhance fire resistance and thermal stability [22,25].

The existing literature provides limited information on the integration of BSG into a PU matrix. Formela et al. [26] conducted experiments on reinforcing PU foam with BSG and ground tire rubber. Results showed that both waste fillers might be used as cheap and environment-friendly reinforcement phase for PU foam. Hejna et al. [27] investigated rigid polyurethane-polyisocyanurate (PUR-PIR) foams incorporating BSG, examining the influence of the isocyanate index on their performance. The compressive performance of the material experienced a deterioration, potentially linked to the increased friability of the highly crosslinked cellular structure in PUR-PIR foams. Variations in the isocyanate index did not significantly affect the quantity or nature of thermal degradation products in the analyzed bio-composites.

Coffee grounds, generated in large quantities by coffee shops and households, possess a unique chemical composition and porous structure [28]. Spent coffee grounds (SCG), similar to brewers’ spent grain, can be repurposed in various ways. They can be used as a soil amendment in agriculture due to their nitrogen content [29]. Additionally, SCG can be processed for biofuel production and have been explored as a component in composite materials [30]. Research indicates that SCG can be transformed into value-added products, including food additives, polyhydroxyalkanoates, carotenoids, biosorbents, activated carbons, polyols, phenolic antioxidants, and nutraceuticals [31]. The incorporation of SCG into polymer technology has the potential to improve mechanical properties, thermal insulation, and fire-retardant capabilities [32,33].

The utilization of SCG as a filler material in PU technology remains limited. Prior applications have primarily focused on SCG as a polyol source [34,35,36]. Auguścik-Królikowska et al. [37] conducted a study on viscoelastic PU foams incorporating coffee ground fillers. The inclusion of 20% filler by mass resulted in a decrease in the rate of heat release and smoke release during foam combustion. Funabashi et al. examined the impact of incorporated plant-derived particles, such as coffee grounds and ground coffee bean parchment, on the mechanical properties of RPUF [38]. Hatakeyama and Hatakeyama reported that incorporating SCG as a filler in lignin polyol-derived polyurethane resulted in enhanced flexural strength and modulus, correlating with increased filler content while maintaining constant density [39]. Bartczak et al. conducted a study on the effectiveness of employing coffee grounds and oak sawdust as fillers in RPUF. Their research indicated that an increased concentration of coffee additives positively influences compressive strength. Furthermore, combustion behavior tests revealed that the incorporation of renewable materials does not adversely affect the fire resistance of the resulting foams [2].

Soybean husks, an agricultural residue from soybean processing, are primarily composed of cellulose, hemicellulose, and lignin. Soybean husks can be utilized as animal feed and also can serve as a raw material for producing biofuels and biopolymers [40]. Moreover, soybean husks can be used to create composite polymeric materials, enhancing their mechanical properties, foam’s structural integrity and thermal characteristics. There is limited information available on the use of soybean husk and soybean husk ash in PU foams. As previously reported, soybean husk has been explored in PU technology as a biopolyol precursor [11,41,42]. Ganesan et al. [17] utilized soy hulls to produce biochar, which was subsequently integrated into flexible PU foam. The introduction of biochar served to sustain the compression properties of the foams at a decreasing isocyanate index, thereby diminishing the necessity for isocyanates in the production process. Furthermore, the incorporation of biochar led to a reduction in the flammability of the foams.

This research investigates the flammability and thermal characteristics of RPUF modified with waste biomass (brewers’ spent grain, coffee grounds, soybean husk) and ash from its combustion. The study aims to assess the effects of incorporating these waste-derived materials on the fire resistance, thermal stability, and insulation properties of the resulting foams. These properties of PU foams are crucial when referring to their application as thermal insulating materials. Moreover, the utilization of the mentioned biomass fillers in PU technology has been limited. To the best of our knowledge, neither a mix of these fillers nor mixed ashes from their combustion have been implemented in RPUF. The application of mixed fillers would more accurately mimic biomass waste management and the production of ash in industrial settings, where multiple waste sources are often combusted together.

## 2. Materials and Methods

### 2.1. Preparation of PU Foam Composites

In this research, RPUF composites were produced utilizing the EKOPRODUR PM4032 commercial system (PCC Group, Brzeg Dolny, Poland; specified product characteristics available online [43]), a two-component system, via a mixing and casting method. The pristine foam system comprised two constituents: an isocyanate (polymeric methylene diphenyl diisocyanate, PMDI) and a polyol premix which contained a petrochemical polyol base, a porogen, and flame-retardant additives.

Six distinct modifiers were integrated into the PU foam for this investigation:

- Ground brewers’ spent grain (labeled B);
- Spent coffee grounds (labeled C);
- Ground soybean husk (labeled S);
- Brewers’ spent grain bottom ash (labeled Ba);
- Spent coffee grounds bottom ash (labeled Ca);
- Soybean husk bottom ash (labeled Sa).

The ash was produced under controlled laboratory conditions, adhering to the EN ISO 18122:2016 standard [44].

Composite RPUF samples were produced by incorporating specified quantities (5% wt.) of biomass waste and the resulting bottom ash into the polyol components, with the amount of filler added determined based on previous work by the research team [45,46,47]. Additionally, two samples were prepared with mixed fillers, consisting of three types of biomass waste (labeled BIO) and three types of biomass waste-derived bottom ash (labeled BIOa), both in the proportion of 1:1:1 by weight. The polyol and filler mixture was thoroughly agitated using a laboratory stirrer at a high speed of 2500 rpm for one minute to achieve homogeneity. Subsequently, the polyol and isocyanate components were combined (weight ratio of 100:120) using the same stirring method for an additional minute before being poured into rectangular molds measuring 20 × 20 × 5 cm. The filled molds were then placed under a fume hood to facilitate setting and curing. Following a 24-h period, the foams were removed from the molds and left to rest under the fume hood for 10 days to ensure the complete removal of any unreacted isocyanate and to stabilize the PU cellular structure. The resulting composite foams were designated as detailed in Table 1, with the unmodified RPUF labeled as PU.

### 2.2. Assessment and Characterization of the Foam Specimens

Elemental composition analysis of the synthesized materials was conducted utilizing a LECO CHN628 Series analyzer (LECO Corporation, St. Joseph, MI, USA) to quantify carbon, hydrogen, and nitrogen content. The gross calorific values of the foam samples were determined using a LECO Isoperibol Calorimeter AC500 (LECO Corporation, St. Joseph, MI, USA). The measurement adhered to the ISO 1716:2018 standard [48].

Fire testing was performed using the cone calorimeter method (Fire Testing Technology Ltd., East Grinstead, UK), in accordance with ISO 5660:2015 [49]. Samples measuring 100 × 100 × 30 mm were exposed to a radiant heat flux of 35 kW m^−2^. The time to ignition, heat release rate, total heat released, effective heat of combustion, total smoke production, and smoke production rate were recorded. Test specimens were arranged in the designated stainless steel holder and backed with a low-density (65 kg m^−3^) ceramic wool fiber blanket, in accordance with the ISO 5660 standard [49]. During the test, test specimens are placed on a calibrated and tared balance for mass loss measurements. The fire effluent sample was taken directly from the exhaust duct and passed through NDIR analyzers for carbon dioxide CO_2_ concentration measurements and paramagnetic analyzers to assess oxygen depletion. Smoke density was determined using a red laser [50].

The thermal conductivity coefficients were determined at room temperature using the Hot Disk TPS 3500 Thermal Constants Analyzer (Hot Disk AB, Goteborg, Sweden), adhering to ISO 2207-2 standards [51]. Prior to assessment, samples measuring 50 × 50 × 30 mm underwent conditioning at a controlled environment of 23 ± 2 °C and 50 ± 5% relative humidity for a minimum duration of 48 h. For each sample type, five individual measurements were conducted, and the results are presented as the average of these five tests along with the standard deviation (SD).

To assess the thermal stability of the materials, thermogravimetric analysis (TG/DTG/DTA) was conducted using Mettler Toledo TGA/DSC 3+ (Mettler Toledo, Greifensee, Switzerland) according to ISO 11358-1 standard [52]. Approximately 4–5 mg samples were analyzed in ceramic pans under a nitrogen atmosphere, with a temperature range of 50–850 °C and a heating rate of 10 °C min^−1^. The temperatures associated with weight loss of 5% (T_5%_)), 10% (T_10%_), and 50% (T_50%_), along with the residual masses at 600 and 800 °C, were determined. The temperatures at which the mass loss rate peaked (T_DTGmax_) were also determined to further elucidate the degradation kinetics and thermal decomposition characteristics.

## 3. Results and Discussion

### 3.1. Fire Resistance Evaluation

The results of the elemental composition analysis (nitrogen, carbon, and hydrogen content), together with the gross calorific values (Q_s_) and thermal conductivity (λ), are detailed in Table 2.

The nitrogen content was relatively consistent across all samples, registering at approximately 7%. A similar trend was observed for hydrogen content, which remained around 11%. The incorporation of ash derived from biomass combustion resulted in a marginal decrease in both N and H content. A parallel trend was noted for carbon content, with foams containing ground biomass exhibiting approximately 70%. The addition of ash, acting as an inert material, to the polyurethane foams led to a slight reduction of about 2% in organic C content. The observed changes were of limited significance, attributable to the relatively low filler content. The gross calorific value also experienced an impact due to the ash addition, with samples containing bottom ash displaying slightly lower values. This reduction is primarily attributed to the non-combustible nature of ash, which effectively dilutes the organic components responsible for caloric release upon combustion. Conversely, the introduction of ground biomass, which consists of organic matter, slightly elevated the calorific values compared to the control polyurethane foam.

The thermal conductivity values of the analyzed samples fell within a narrow range of 34–37 mW m^−1^K^−1^. The low standard deviation values, not exceeding 1% of λ values, indicated a high degree of uniformity in the composite foams at the macroscopic level. Given the relatively low filler content (5% wt.), the alteration in thermal conductivity in relation to the unmodified PU foam was not substantial. The determined λ values aligned with those reported in the literature for polyurethane foam composites [53,54,55]. The consistent thermal conductivity across samples suggests that the various biomass fillers, at the concentrations used, did not significantly perturb the insulating properties inherent to rigid polyurethane foam [37]. In comparison to conventional thermal insulation materials, all fabricated RPUF samples exhibited lower thermal conductivity values than those recorded for mineral wool (40 mW m^−1^K^−1^) and rock wool (45 mW m^−1^K^−1^) [1], and were comparable to those reported for expanded polystyrene (EPS; 35 mW m^−1^K^−1^) [56].

### 3.2. Cone Calorimeter Analysis

Cone calorimeter tests were conducted to comprehensively evaluate the flammability characteristics of the modified rigid polyurethane foams, providing insights into parameters such as: time to ignition (TIG), maximum heat release rate (pekHRR), average heat release rate (meanHRR), total heat release (THR), maximum effective heat of combustion (peakEHC), total smoke production (TSP), total smoke release (TSR), and mass loss (Δm). The data gathered during the experiment is presented in Table 3.

The TIG was similar across nearly all samples, suggesting that the modifications did not substantially influence the foams’ flammability at this initial stage. However, the incorporation of fillers in the PU+BIO and PU+Ba samples notably delayed ignition. Generally, biomass heats more slowly than the polyurethane matrix, requiring a longer duration to reach the temperature necessary for ignition [57]. The peakHRR values for samples containing ground biomass were similar to those of the reference sample. However, the inclusion of biomass fillers led to a slight increase in this parameter, which is associated with the slower heating of the biomass compared to the polymer matrix. Samples containing bottom ash exhibited the lowest values, with the PU+Ba composite demonstrating the lowest peak heat release rate. The presence of ash particles, located between the polyurethane cells, impeded the release rate of volatile gases and the penetration of oxygen into the burning foam [17]. Foam containing mixed bottom ash showed a peak heat release rate comparable to that of the reference sample.

The meanHRR varied between 11 and 36 kW m^−2^, with no discernible trend. However, lower values were generally observed for ash-containing foams. The presence of ash, a non-combustible filler, act as a barrier, reducing the heat release rate during combustion [9]. The THR was reduced in samples incorporating biomass fillers. The introduction of ash also resulted in a decrease in this parameter, although to a lesser extent. These findings are consistent with the previously established conclusions regarding alterations in peak and mean heat release rates. The peakEHC, a key parameter directly related to flammability, indicates the amount of heat a material releases during combustion. The maximum effective heat of combustion was similar across all samples, suggesting that none of the fillers significantly affected the polyurethane foam’s flammability. Minor decreases in this parameter did not exceed 1% of the reference value measured for PU. The inclusion of ashes led to a reduction in both TSP and TSR, indicating their efficacy as smoke suppressants [9,58,59]. The Δm was found to be similar for all samples, indicating that the fillers did not significantly affect the amount of material consumed during combustion.

**Figure 1:**
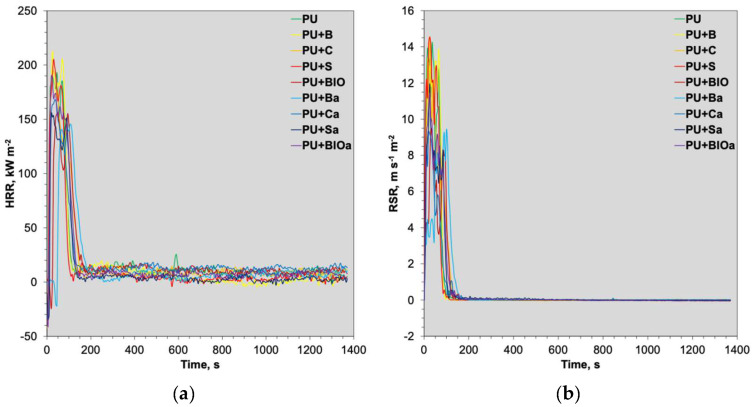
Temporal dynamics of heat release rate, HRR (**a**) and rate of smoke release, RSR (**b**) of RPUF composites during cone calorimeter testing.

Figure 1 illustrates the temporal variations in heat release rate (HRR) and rate of smoke release (RSR). The highest peak of the HRR curves was obtained for unmodified foam (PU) and samples containing ground biomass (PU+B, PU+C, and PU+S), reaching 195–215 kW m^−2^. A similar result was also observed for the sample containing mixed ash (PU+BIOa; approximately 190 kW m^−2^). All the other materials, containing individual ashes and mixed biomass, expressed lower profiles, reaching maximum values of 150–170 kW m^−2^. These findings indicate that ash particles act as a barrier, which decreases the heat release rate during combustion. All the differences between samples were visible during the first 200 s of analysis. After this time, curves overlap each other, indicating a similar heat release rate for all the samples. The RSR exhibited a similar trend, with the highest peaks observed for samples containing ground biomass and the reference sample. Conversely, the lowest values were recorded for samples containing individual ashes and mixed biomass.

**Figure 2:**
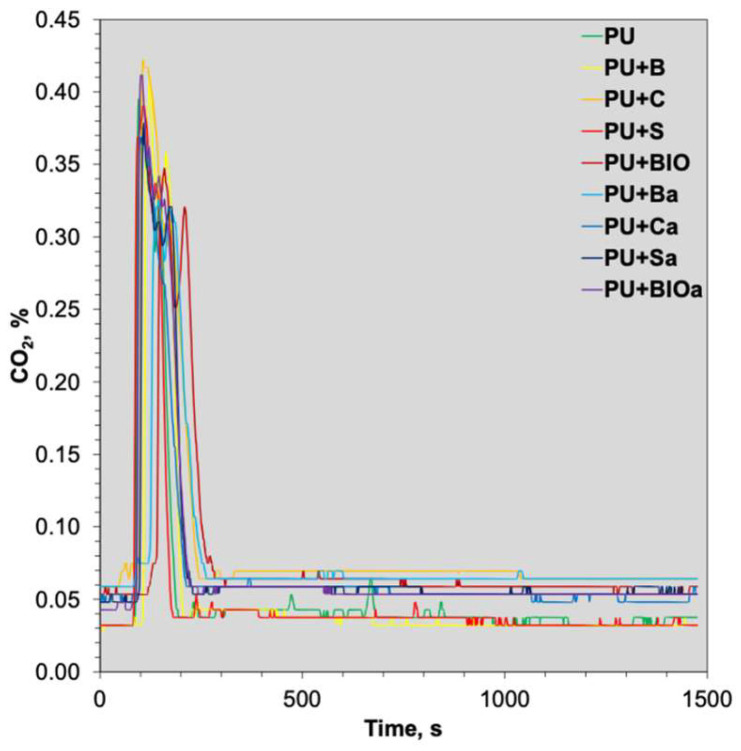
Temporal dynamics of carbon dioxide, CO_2_ emissions of RPUF composites during cone calorimeter testing.

Figure 2 illustrates the emission of asphyxiant combustion gas (carbon dioxide CO_2_ over time. Given the open combustion chamber setup, the resulting carbon monoxide CO concentrations were minimal, measured in ppm. Consequently, the emission of this particular flue gas was not quantified. The release of CO_2_ was comparable for the reference material and almost all modified samples. Only two samples, PU+BIO and PU+Ba, exhibited slightly lower emissions and marginally shifted emission profiles; however, these changes were not significant. After 300 s of analysis, all the emission profiles were similar. Some samples (containing ashes, coffee grounds and mixed biomass) exhibited slightly higher CO_2_ emission baselines, but the difference remained within the margin of measurement error.

### 3.3. Thermal Properties Investigation

**Figure 3:**
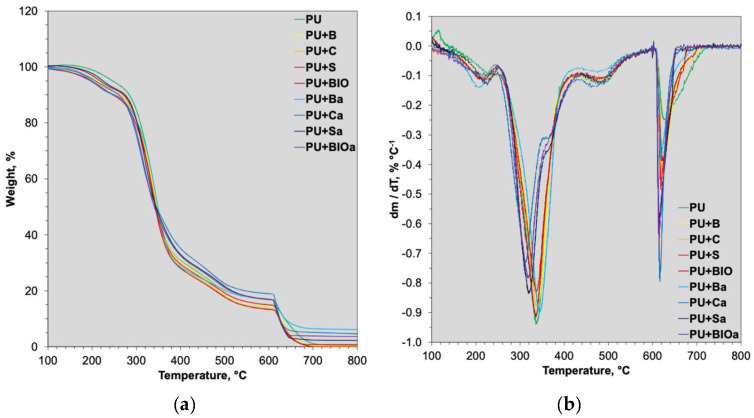
TG (**a**), and DTG (**b**) curves of RPUF composites.

The thermal stability of both unmodified PU foam and the composites produced with added fillers was investigated using thermogravimetric analysis. Figure 3 presents the thermogravimetric (TG) and differential thermogravimetric curves (DTG), while Table 4 summarizes the thermal stability parameters derived from the TG/DTG curves.

The highest onset temperature for degradation T_5%_ (247 °C) was observed in pristine PU foam, indicating that the additives tend to lower this temperature. The lowest T_5%_ was recorded for PU+BIOa (196 °C). Similarly, T_10%_ and T_10%_ followed the same trend, with unmodified sample exhibiting the highest values, while the additives generally resulted in reduced temperatures. This suggests that while the biomass and ash may influence flammability characteristics, they do not inherently improve the intrinsic thermal stability of the polyurethane matrix itself [60]. The pristine rigid polyurethane foam demonstrates the highest onset temperatures, indicating its resistance to initial thermal decomposition. This resilience is attributed to the absence of fillers that could accelerate bond scission; thus, degradation occurs predominantly through the cleavage of urethane and polyol linkages at elevated temperatures [61,62]. In contrast, the inclusion of biomass and ash, even at low concentrations, introduces heterogeneity within the polymer matrix, providing potential sites for accelerated thermal degradation [63,64].

In the case of residue at 600 and 800 °C, the highest char yields (approximately 17 and 5%, respectively) were consistently observed in samples containing ash, suggesting their effectiveness in promoting char formation, which is a crucial mechanism for enhancing flame retardancy [65]. Both unmodified foam and composites containing ground biomass, exhibited similar low char yields (approximately 14 and 1% at 600 and 800 °C, respectively), indicating that these additives did not significantly contribute to char formation.

The disparate charring efficacy between raw biomass and its combustion ashes in rigid polyurethane foams can be attributed to chemical and structural transformations occurring during the incineration process. Raw biomass, primarily composed of organic polymers like cellulose, hemicellulose, and lignin, undergoes devolatilization and pyrolysis at lower temperatures, leading to the release of combustible gases and a relatively small, often unstable, char residue [66]. Conversely, the combustion ashes are predominantly inorganic, composed of metal oxides and silicates, which are inherently non-combustible and possess a stable, porous structure that can act as a physical barrier and promote char formation at elevated temperatures [18]. This distinction is critical as the inorganic constituents in biomass ash, such as calcium carbonate, can undergo endothermic decomposition, releasing CO_2_ and diluting the flame zone, thereby contributing to local cooling and enhanced char integrity [9]. Furthermore, the catalytic activity of certain metal oxides present in ash can facilitate the cross-linking and aromatization of the polymer matrix, leading to a more robust and denser char layer compared to the less stable char formed from raw biomass [9].

The thermal decomposition of rigid polyurethane foams typically occurs in multiple stages, reflecting the degradation of various polymeric segments and incorporated additives [67]. Specifically, the initial degradation phase often involves the cleavage of urethane linkages and the volatilization of residual low molecular weight compounds, while subsequent stages correspond to the breakdown of the soft and hard segments of the polymer matrix [37]. The introduction of fillers, such as biomass and ash, can significantly alter these degradation profiles by acting as physical barriers, char-forming agents, or diluting the combustible material [35,37].

Thermogravimetric analysis indicated that all samples underwent a two-stage degradation process. The initial phase, observed approximately between 200 and 400 °C, is predominantly attributed to the decomposition of rigid segments. The subsequent phase, typically occurring between 500 and 700 °C, is linked to the further degradation of the flexible polyol segments. This multi-stage decomposition pattern is characteristic of segmented polyurethanes, where the thermal stability of the hard segments (urethane linkages) is generally lower than that of the soft segments (polyol chains) [27].

The temperatures at which the mass loss rate reached its maximum for each stage of degradation were determined, offering insight into the kinetics of the decomposition process. For both the unmodified foam and those modified with ground biomass, the T_DTGmax1_ and T_DTGmax2_ values were comparable. However, foams incorporating ashes displayed lower T_DTGmax1_ and T_DTGmax2_ values, indicating that ash presence accelerated the decomposition process. This suggests a catalytic influence of the inorganic ash constituents on the thermal breakdown of the polyurethane matrix, possibly due to their acidity or surface area that promotes bond scission [37]. This finding is consistent with prior research demonstrating that specific inorganic fillers can reduce the activation energy for polymer degradation by providing active sites for thermal scission or by modifying the diffusion pathways of volatile products [68].

**Figure 4:**
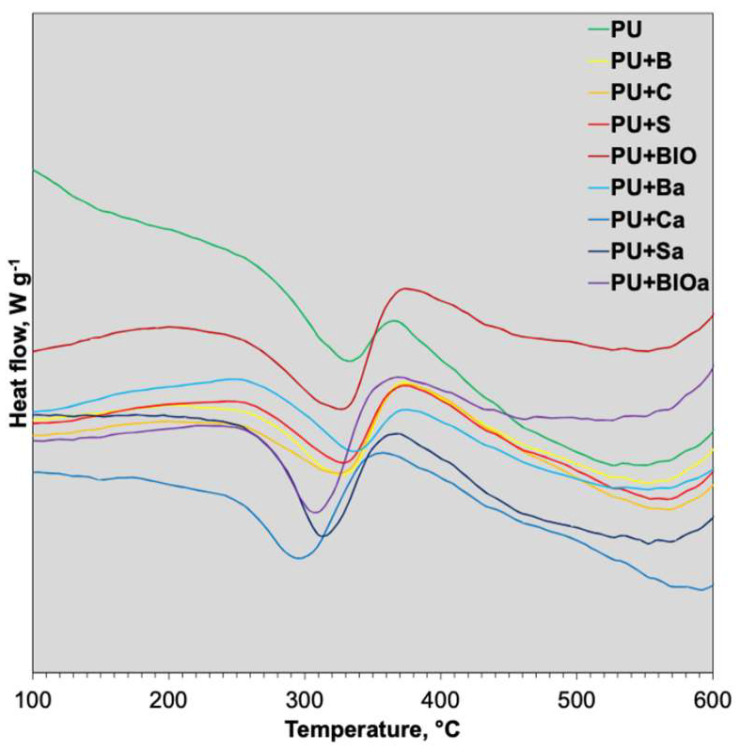
DTA curves of RPUF composites.

Differential thermal analysis was conducted to further elucidate the thermal properties of the materials, with the resulting curves presented in Figure 4 and summarized data in Table 5.

The DTA profiles for the analyzed polyurethane foams exhibited a prominent peak near 300 °C, indicative of the primary thermal decomposition phase, characterized by the breakdown of urethane bonds and partial decomposition of the polyol backbone. While the incorporation of ash did not alter the onset decomposition temperatures, it led to a reduction in the peak decomposition temperature, thereby diminishing the overall thermal stability. A general decrease in the specific enthalpy change was also observed with ash addition. This phenomenon can be attributed to the largely inert nature of ash, which does not undergo significant enthalpy changes within the tested temperature range, and the reduced proportion of combustible polymer per unit mass of the composite, leading to a lower measured heat release from polymer degradation/oxidation [69,70]. Additionally, endothermic decomposition reactions of certain ash components might partially counteract the exothermic heat generated during polymer combustion, and ash minerals could catalyze the breakdown of polymers into volatile products that escape without substantial heat release.

## 4. Conclusions

This comprehensive analysis provides critical insights into the complex interplay between waste biomass, ash, and the thermal and flammability characteristics of rigid polyurethane foams. RPUF are excellent insulators but inherently pose a significant flammability challenge. Research and modification efforts, particularly with materials like combustion ash, aim to mitigate these flammability concerns by enhancing char formation and reducing heat release, though these modifications do not necessarily improve the foam’s intrinsic thermal stability or may even slightly reduce it. Future research should therefore focus on optimizing the synergistic effects between biomass-derived fillers and inorganic ash components to develop more effective flame-retardant rigid polyurethane foams. Further investigations could explore the surface functionalization of these waste materials to enhance their compatibility with the polymer matrix and to introduce additional flame-retardant mechanisms. Furthermore, the utilization of polyols with lower densities could facilitate the inclusion of greater quantities of these additives, thereby more effectively impacting thermal insulation characteristics while preserving adequate mechanical strength.

What is more, in order to improve the intrinsic thermal stability of RPUF without compromising its insulation properties, several strategies can be employed. While the incorporation of certain lignocellulosic biomass has been shown to enhance the thermal stability of reinforced polyurethane foams, others exhibit no significant influence [71]. This suggests that the type of biomass filler plays a crucial role in its effect on thermal stability. Specifically, the chemical composition of the biomass, particularly the proportion of cellulose, hemicellulose, and lignin, along with its physical characteristics like particle size and morphology, dictates its interaction with the polyurethane matrix and its char-forming potential [71]. Moreover, the presence of specific inorganic components in biomass ash, such as phosphates or silicates, can act as fire retardants by forming a protective char layer or diluting combustible gases, thereby further improving the overall thermal stability of the composite [68]. Future investigations should therefore explore a wider array of biomass waste products as potential fillers, taking into account their distinct chemical compositions. Additionally, both heterocyclic-containing polyols [72] and silicon-based fillers, including colloidal silica and polyhedral oligomeric silsesquioxane (POSS) [73,74,75], effectively enhanced the inherent thermal stability of rigid polyurethane foams. The combination of these materials can lead to RPUFs with superior thermal resistance and flame retardancy, making them suitable for applications requiring high thermal stability. The utilization of the aforementioned methodologies presents a viable pathway for the creation of sustainable and high-performance flame-retardant materials derived from industrial byproducts, thereby reinforcing the tenets of circular economy and green chemistry principles.

While emphasizing circular economy principles and a life-cycle perspective, it is essential to acknowledge the inherent trade-offs accompanying the integration of ash into RPUF. Variations in the chemical composition and particle dimensions of waste-derived ash necessitate pre-use characterization, potentially incurring additional testing and processing expenses. Moreover, the logistical complexities and associated costs of collecting, transporting, and processing these waste materials, particularly when sourced from disparate locations, cannot be overlooked. The energy demands of processing biomass waste into usable fillers might also mitigate some of the anticipated environmental advantages. Consequently, ensuring the overall environmental sustainability of the filler’s lifecycle, from procurement to final disposal, is paramount to preclude adverse ecological consequences. In summation, while the incorporation of ash into RPUF yields notable improvements in fire resistance and contributes to environmental sustainability, these benefits are counterbalanced by potential economic and environmental costs tied to sourcing, processing, and integration. A considerate balancing of these competing factors necessitates a comprehensive evaluation of the materials’ entire lifecycle.

## Figures and Tables

**Table 1:** Nomenclature and composition of foam samples.

Sample Name	Filler					
	Brewers’ Spent Grain (B)	Coffee Grounds (C)	Soybean Husk (S)	Brewers’ Spent Grain Ash (Ba)	Coffee Grounds Ash (Ca)	Soybean Husk Ash (Sa)
PU						
PU+B	✕					
PU+C		✕				
PU+S			✕			
PU+BIO	✕	✕	✕			
PU+Ba				✕		
PU+Ca					✕	
PU+Sa						✕
PU+BIOa				✕	✕	✕

**Table 2:** Elemental composition, gross calorific values (Q_s_) and thermal conductivity (λ) of obtained materials.

Sample Name	N, % wt.	C, % wt.	H, % wt.	Q_s_, kJ g^−1^	λ, mW m^−1^K^−1^
PU	6.56	69.45	10.86	26.14	34.93 ± 0.07
PU+B	6.73	69.07	10.93	26.69	34.83 ± 0.01
PU+C	6.84	69.63	10.99	26.45	35.20 ± 0.02
PU+S	7.22	71.03	11.08	26.51	33.92 ± 0.03
PU+BIO	6.98	69.15	11.11	26.74	34.79 ± 0.01
PU+Ba	5.71	64.47	11.14	25.10	37.37 ± 0.03
PU+Ca	6.82	67.51	10.46	25.82	36.12 ± 0.34
PU+Sa	6.80	67.79	10.40	26.02	34.97 ± 0.04
PU+BIOa	6.62	66.65	10.28	25.90	34.88 ± 0.03

**Table 3:** Cone calorimeter test results of RPUF composites.

Sample Name	TIG, s	PeakHRR, kW m^−2^	MeanHRR, kW m^−2^	THR, MJ m^−2^	PeakEHC, MJ kg^−1^	TSP, m^2^	TSR, m^2^ m^−2^	Δm, –
PU	4	193.0	16.9	30.4	79.8	7.9	890.3	10.2
PU+B	3	212.8	36.1	20.5	74.1	8.0	908.0	9.8
PU+C	5	194.9	28.6	22.4	76.7	8.1	914.7	13.4
PU+S	2	205.1	11.4	20.7	77.4	7.5	845.4	10.0
PU+BIO	21	156.2	26.3	21.1	79.4	5.2	585.0	9.7
PU+Ba	45	145.4	13.4	24.2	78.7	5.3	602.9	9.1
PU+Ca	4	167.9	19.5	35.2	77.3	5.0	564.0	8.6
PU+Sa	7	155.8	10.6	19.3	71.8	6.7	761.1	11.4
PU+BIOa	3	189.9	15.9	28.7	77.5	7.3	825.8	11.8

**Table 4:** Thermal degradation characteristics of RPUF composites from TG/DTG curves.

Sample Name	T_5%_, °C	T_10%_, °C	T_50%_, °C	T_DTGmax1_, °C	T_DTGmax2_, °C	Residue at 600 °C, %	Residue at 800 °C, %
PU	247	284	347	335	626	15.07	0.59
PU+B	211	263	347	340	623	14.85	0.23
PU+C	206	264	345	337	621	13.75	0.18
PU+S	200	247	344	335	618	15.03	0.77
PU+BIO	218	271	343	335	622	13.42	0.16
PU+Ba	200	248	349	343	619	16.98	6.17
PU+Ca	208	260	345	311	617	19.03	4.60
PU+Sa	225	274	344	319	614	16.98	2.26
PU+BIOa	196	250	341	317	614	17.09	3.68

**Table 5:** Thermal characteristics of RPUF composites from DTA curves.

Sample Name	Onset Temperature, °C	Peak Temperature, °C	Specific Enthalpy Change, J g^−1^
PU	285	334	−104
PU+B	273	334	−156
PU+C	267	333	−149
PU+S	269	334	−133
PU+BIO	253	334	−259
PU+Ba	286	340	−85
PU+Ca	250	302	−196
PU+Sa	282	318	−172
PU+BIOa	272	315	−180

## Data Availability

The original contributions presented in the study are included in the article, further inquiries can be directed to the corresponding author.

## Abbreviations

The following abbreviations are used in this manuscript:

EPS	Expanded polystyrene
RPUF	Rigid polyurethane foam
PU	Polyurethane
BSG	Brewers’ spent grain
SCG	Spent coffee grounds
PMDI	Polymeric methylene diphenyl diisocyanate
SD	Standard deviation
TG	Thermogravimetric analysis
DTG	Derivative thermogravimetry
DTA	Differential thermal analysis
POSS	Polyhedral oligomeric silsesquioxane

## References

[1] Jeon C.K., Lee J.S., Chung H., Kim J.H., Park J.P. (2017). A study on insulation characteristics of glass wool and mineral wool coated with a polysiloxane agent. Adv. Mater. Sci. Eng..

[2] Bartczak P., Stachowiak J., Szmitko M., Grząbka-Zasadzińska A., Borysiak S. (2023). Multifunctional Polyurethane Composites with Coffee Grounds and Wood Sawdust. Materials.

[3] Qian L., Li L., Chen Y., Xu B., Qiu Y. (2019). Quickly self-extinguishing flame retardant behavior of rigid polyurethane foams linked with phosphaphenanthrene groups. Compos. Part B.

[4] Michálková D., Ďurica P. (2022). Experimental Verification of Thermal Insulation in Timber Framed Walls. Materials.

[5] Del Saz-Orozco B., Alonso M.V., Oliet M., Domínguez J.C., Rojo E., Rodriguez F. (2015). Lignin particle-and wood flour-reinforced phenolic foams: Friability, thermal stability and effect of hygrothermal aging on mechanical properties and morphology. Compos. Part B.

[6] Lisowski P., Glinicki M.A. (2025). Promising biomass waste–derived insulation materials for application in construction and buildings. Biomass Convers. Biorefin..

[7] Malchiodi B., Marchetti R., Barbieri L., Pozzi P. (2022). Recovery of Cork Manufacturing Waste within Mortar and Polyurethane: Feasibility of Use and Physical, Mechanical, Thermal Insulating Properties of the Final Green Composite Construction Materials. Appl. Sci..

[8] Yang Y., Haurie L., Wang D.Y. (2022). Bio-based materials for fire-retardant application in construction products: A review. J. Therm. Anal. Calorim..

[9] Kairytė A., Kremensas A., Vaitkus S., Członka S., Strąkowska A. (2020). Fire Suppression and Thermal Behavior of Biobased Rigid Polyurethane Foam Filled with Biomass Incineration Waste Ash. Polymers.

[10] Hejna A. (2021). Clays as Inhibitors of Polyurethane Foams’ Flammability. Materials.

[11] Chi J., Zhang Y., Tu F., Sun J., Zhi H., Yang J. (2023). The synergistic flame-retardant behaviors of soybean oil phosphate-based polyols and modified ammonium polyphosphate in polyurethane foam. J. Polym. Res..

[12] Song F., Jia P., Bo C., Ren X., Hu L., Zhou Y. (2020). The mechanical and flame retardant characteristics of lignin-based phenolic foams reinforced with MWCNTs by in-situ polymerization. J. Dispers. Sci. Technol..

[13] Widsten P., Tamminen T., Paajanen A., Hakkarainen T., Liitiä T. (2021). Modified and unmodified technical lignins as flame retardants for polypropylene. Holzforschung.

[14] Zhou K., Gong K., Zhou Q., Zhao S., Guo H., Qian X. (2020). Estimating the feasibility of using industrial solid wastes as raw material for polyurethane composites with low fire hazards. J. Clean. Prod..

[15] Väisänen T., Das O., Tomppo L. (2017). A review on new bio-based constituents for natural fiber-polymer composites. J. Clean. Prod..

[16] Agrawal A., Kaur R., Walia R.S. (2019). Investigation on flammability of rigid polyurethane foam-mineral fillers composite. Fire Mater..

[17] Ganesan K., Guin B., Wilbanks E., Sternberg J. (2025). Synthesis and Characterization of Soy Hull Biochar-Based Flexible Polyurethane Foam Composites. Materials.

[18] Miedzianowska-Masłowska J., Masłowski M., Strzelec K. (2025). Biomass, Phyto-Ash, and Biochar from Beech Wood as Functional Additives for Natural Rubber-Based Elastomer Composites. Materials.

[19] Wrześniewska-Tosik K., Ryszkowska J., Mik T., Wesołowska E., Kowalewski T., Pałczyńska M., Sałasińska K., Walisiak D., Czajka A. (2020). Composites of Semi-Rigid Polyurethane Foams with Keratin Fibers Derived from Poultry Feathers and Flame Retardant Additives. Polymers.

[20] Lin L., Mirkin S., Park H.E. (2023). Biodegradable Composite Film of Brewers’ Spent Grain and Poly(Vinyl Alcohol). Processes.

[21] Salman W., Ney Y., Nasim M.J., Bohn T., Jacob C. (2020). Turning apparent waste into new value: Up-cycling strategies exemplified by Brewer’s spent grains (BSG). Curr. Nutraceuticals.

[22] Pérocheau Arnaud S. (2024). Valorisation of Brewer’s Spent Grain: Lignocellulosic Fractionation and its Potential for Polymer and Composite Material Applications. Chem. Afr..

[23] Chetrariu A., Dabija A. (2020). Brewer’s Spent Grains: Possibilities of Valorization, a Review. Appl. Sci..

[24] Jackowski M., Niedźwiecki Ł., Jagiełło K., Uchańska O., Trusek A. (2020). Brewer’s Spent Grains—Valuable Beer Industry By-Product. Biomolecules.

[25] Hejna A., Barczewski M., Skórczewska K., Szulc J., Chmielnicki B., Korol J., Formela K. (2021). Sustainable upcycling of brewers’ spent grain by thermo-mechanical treatment in twin-screw extruder. J. Clean. Prod..

[26] Formela K., Hejna A., Zedler Ł., Przybysz M., Ryl J., Saeb M.R., Piszczyk Ł. (2017). Structural, thermal and physico-mechanical properties of polyurethane/brewers’ spent grain composite foams modified with ground tire rubber. Ind. Crops Prod..

[27] Hejna A., Haponiuk J., Piszczyk Ł., Klein M., Formela K. (2017). Performance properties of rigid polyurethane-polyisocyanurate/brewers’ spent grain foamed composites as function of isocyanate index. E-Polymers.

[28] Thomas B.S., Yang J., Mo K.H., Abdalla J.A., Hawileh R.A., Ariyachandra E. (2021). Biomass ashes from agricultural wastes as supplementary cementitious materials or aggregate replacement in cement/geopolymer concrete: A comprehensive review. J. Build. Eng..

[29] Stufano P., Perrotta A., Labarile R., Trotta M. (2022). The second life of coffee can be even more energizing: Circularity of materials for bio-based electrochemical energy storage devices. MRS Energy Sustain..

[30] Johnson K., Liu Y., Lu M. (2022). A review of recent advances in spent coffee grounds upcycle technologies and practices. Front. Chem. Eng..

[31] Karmee S.K. (2018). A spent coffee grounds based biorefinery for the production of biofuels, biopolymers, antioxidants and biocomposites. Waste Manag..

[32] Xiao Z.H., Hou X.Q., Hwang S.S., Li H.M. (2022). The biocomposites properties of compounded poly (lactic acid) with untreated and treated spent coffee grounds. J. Appl. Polym. Sci..

[33] Nguyen T.A., Nguyen Q.T. (2021). Hybrid biocomposites based on used coffee grounds and epoxy resin: Mechanical properties and fire resistance. Int. J. Chem. Eng..

[34] Paria S., Kim G., Lee J.W., Jeong S., Sahu P., Park S.H., Oh J.S. (2024). Ecopolyols from Spent Coffee Grounds Through Acid Liquefaction Using Polyol: Synthesis and its Optimization. J. Polym. Environ..

[35] Olszewski A., Kosmela P., Vēvere L., Olszewski A., Kosmela P., Vēvere L., Kirpluks M., Cabulis U., Piszczyk Ł. (2024). Effect of bio-polyol molecular weight on the structure and properties of polyurethane-polyisocyanurate (PUR-PIR) foams. Sci. Rep..

[36] Arias A., Ioannidou S.M., Giannakis N., Feijoo G., Moreira M.T., Koutinas A. (2023). Review of potential and prospective strategies for the valorization of coffee grounds within the framework of a sustainable and circular bioeconomy. Ind. Crops Prod..

[37] Auguścik-Królikowska M., Ryszkowska J., Ambroziak A., Szczepkowski L., Oliwa R., Oleksy M. (2020). The structure and properties of viscoelastic polyurethane foams with fillers from coffee grounds. Polimery.

[38] Funabashi M., Hirose S., Hatakeyama T., Hatakeyama H. (2003). Effect of filler shape on mechanical properties of rigid polyurethane composites containing plant particles. Macromol. Symp..

[39] Hatakeyama H., Hatakeyama T., Abe A., Dusek K., Kobayashi S. (2009). Lignin Structure, Properties, and Applications. Biopolymers.

[40] Biazatti M.J., de Carvalho Miranda J.C. (2021). Soybean-based concept biorefinery. Biofuels Bioprod. Biorefin..

[41] Kakarla V.M. (2003). Preparation of Soy Augmented Water Blown Polyurethane Foam and Evaluation of Mechanical Properties. Master’s Thesis.

[42] Ji D., Fang Z., He W., Luo Z., Jiang X., Wang T., Guo K. (2015). Polyurethane rigid foams formed from different soy-based polyols by the ring opening of epoxidised soybean oil with methanol, phenol, and cyclohexanol. Ind. Crops Prod..

[43] EKOPRODUR PM4032 Characteristics Sheet. https://www.products.pcc.eu/wp-content/uploads/import/broszura/2020-05-22/2b322bd8-5c34-4786-bd88-fffce8e829d9/ekoprodur-pm4032_broszura_en.pdf.

[44] (2022). Solid Biofuels—Determination of Ash Content.

[45] Magiera A., Kuźnia M., Jerzak W. (2025). Analysis of the Structural, Chemical, and Mechanical Characteristics of Polyurethane Foam Infused with Waste from Thermal Processing. Materials.

[46] Magiera A., Kuźnia M., Błoniarz A., Magdziarz A. (2023). Rigid Polyurethane Foams Modified with Soybean-Husk-Derived Ash as Potential Insulating Materials. Processes.

[47] Zakrzewska P., Kuźnia M., Zygmunt-Kowalska B., Magiera A., Magdziarz A. (2023). Utilization of Sunflower Husk Ash in the Production of Polyurethane Materials. Energies.

[48] (2018). Reaction to Fire Tests for Products—Determination of the Gross Heat of Combustion (Calorific Value).

[49] (2015). Reaction-to-Fire Tests—Heat Release, Smoke Production and Mass Loss Rate. Part 1: Heat Release Rate (Cone Calorimeter Method) and Smoke Production Rate (Dynamic Measurement).

[50] Kaczorek-Chrobak K., Fangrat J., Papis B.K. (2021). Calorimetric Behaviour of Electric Cables. Energies.

[51] (2022). Plastics—Determination of Thermal Conductivity and Thermal Diffusivity. Part 2: Transient Plane Heat Source (Hot Disc) Method.

[52] (2022). Plastics—Thermogravimetry (TG) of Polymers.

[53] Kairytė A., Kizinievič O., Kizinievič V., Kremensas A. (2019). Synthesis of biomass-derived bottom waste ash based rigid biopolyurethane composite foams: Rheological behaviour, structure and performance characteristics. Compos. Part A.

[54] Członka S., Strąkowska A., Kairytė A., Kremensas A. (2020). Nutmeg filler as a natural compound for the production of polyurethane composite foams with antibacterial and anti-aging properties. Polym. Test..

[55] Członka S., Strąkowska A., Kairytė A. (2020). Effect of walnut shells and silanized walnut shells on the mechanical and thermal properties of rigid polyurethane foams. Polym. Test..

[56] Naldzhiev D., Mumovic D., Strlic M. (2020). Polyurethane insulation and household products–A systematic review of their impact on indoor environmental quality. Build. Environ..

[57] Dukarska D., Mirski R. (2024). Current Trends in the Use of Biomass in the Manufacture of Rigid Polyurethane Foams: A Review. J. Compos. Sci..

[58] Bo G., Xu X., Tian X., Wu J., Yan Y. (2021). Enhancing the Fire Safety and Smoke Safety of Bio–Based Rigid Polyurethane Foam via Inserting a Reactive Flame Retardant Containing P@N and Blending Silica Aerogel Powder. Polymers.

[59] Liu Y., Li H., Chen Q., Luo F., Cao C. (2020). Effect of bamboo flour on flame retardancy and smoke suppression of polypropylene/ammonium polyphosphate composites. Front. Mater..

[60] Bajer K., Kaczmarek H., Dzwonkowski J., Stasiek A., Ołdak D. (2007). Photochemical and thermal stability of degradable PE/paper waste composites obtained by extrusion. J. Appl. Polym. Sci..

[61] Głowacz-Czerwonka D., Zakrzewska P., Zygmunt-Kowalska B., Zarzyka I. (2025). Thermal and Flammability Analysis of Polyurethane Foams with Solid and Liquid Flame Retardants: Comparative Study. Polymers.

[62] Jasiūnas L., McKenna S.T., Bridžiuvienė D., Miknius L. (2020). Mechanical, Thermal Properties and Stability of Rigid Polyurethane Foams Produced with Crude-Glycerol Derived Biomass Biopolyols. J. Polym. Environ..

[63] Otaru A.J., Albin Zaid Z.A.A. (2025). Thermal Degradation of Palm Fronds/Polypropylene Bio-Composites: Thermo-Kinetics and Convolutional-Deep Neural Networks Techniques. Polymers.

[64] Sikora J.W., Majewski Ł., Puszka A. (2021). Modern Biodegradable Plastics—Processing and Properties Part II. Materials.

[65] Mizera K., Sałasińska K., Ryszkowska J., Kurańska M., Kozera R. (2021). Effect of the Addition of Biobased Polyols on the Thermal Stability and Flame Retardancy of Polyurethane and Poly(urea)urethane Elastomers. Materials.

[66] Wang X., Ma D., Jin Q., Deng S., Stančin H., Tan H., Mikulčić H. (2019). Synergistic effects of biomass and polyurethane co-pyrolysis on the yield, reactivity, and heating value of biochar at high temperatures. Fuel Process. Technol..

[67] Reinerte S., Jurkjane V., Cabulis U., Viksna A. (2021). Identification and Evaluation of Hazardous Pyrolysates in Bio-Based Rigid Polyurethane-Polyisocyanurate Foam Smoke. Polymers.

[68] Olejnik A., Kosmela P., Piszczyk Ł. (2021). Enhancement of PUR/PIR foam thermal stability after addition of Zostera marina biomass component investigated via thermal analysis and isoconversional kinetics. J. Polym. Sci..

[69] Dutta S., Kim N.K., Das R., Bhattacharyya D. (2021). Evaluating Orientation Effects on the Fire Reaction Properties of Flax-Polypropylene Composites. Polymers.

[70] Kim Y., Lee S., Yoon H. (2021). Fire-Safe Polymer Composites: Flame-Retardant Effect of Nanofillers. Polymers.

[71] Zhang J., Hori N., Takemura A. (2023). Effect of natural biomass fillers on the stability, degradability, and elasticity of crop straws liquefied polyols-based polyurethane foams. J. Appl. Polym. Sci..

[72] Guo H., Gao Q., Ouyang C., Zheng K., Xu W. (2015). Research on properties of rigid polyurethane foam with heteroaromatic and brominated benzyl polyols. J. Appl. Polym. Sci..

[73] Lee D.I., Ha Y.H., Jeon H., Kim S.H. (2022). Preparation and Properties of Polyurethane Composite Foams with Silica-Based Fillers. Appl. Sci..

[74] Liszkowska J., Czupryński B., Paciorek-Sadowska J. (2013). The influence silica, kaolin and epoxy resin on heat and thermal properties of rigid polyurethane–polyisocyanurate foams. J. Cell. Plast..

[75] Michałowski S., Hebda E., Pielichowski K. (2017). Thermal stability and flammability of polyurethane foams chemically reinforced with POSS. J. Therm. Anal. Calorim..

